# Transmission of paediatric respiratory syncytial virus and influenza in the wake of the COVID-19 pandemic

**DOI:** 10.2807/1560-7917.ES.2021.26.29.2100186

**Published:** 2021-07-22

**Authors:** Thomas C. Williams, Ian Sinha, Ian G. Barr, Maria Zambon

**Affiliations:** 1 MRC Human Genetics Unit, Institute of Genetics and Cancer, University of Edinburgh, Edinburgh, United Kingdom; 2 University of Liverpool, Liverpool, United Kingdom; 3 WHO Collaborating Centre for Reference and Research on Influenza, Doherty Institute, Melbourne, Australia; 4 Virus Reference Department, Public Health England, Colindale, United Kingdom

**Keywords:** Respiratory Syncytial Virus, influenza, respiratory infections

## Abstract

The non-pharmaceutical interventions implemented to slow the spread of SARS-CoV-2 have had consequences on the transmission of other respiratory viruses, most notably paediatric respiratory syncytial virus (RSV) and influenza. At the beginning of 2020, lockdown measures in the southern hemisphere led to a winter season with a marked reduction in both infections. Intermittent lockdowns in the northern hemisphere also appeared to interrupt transmission during winter 2020/21. However, a number of southern and northern hemisphere countries have now seen delayed RSV peaks. We examine the implications of these unpredictable disease dynamics for health service delivery in Europe, such as paediatric hospital and intensive care bed space planning, or palivizumab prophylaxis. We discuss the challenges for RSV vaccine trials and influenza immunisation campaigns, and highlight the considerable research opportunities that have arisen with the SARS-CoV-2 pandemic. We argue that the rapid advances in viral whole genome sequencing, phylogenetic analysis, and open data sharing during the pandemic are applicable to the ongoing surveillance of RSV and influenza. Lastly, we outline actions to prepare for forthcoming influenza seasons and for future implementation of RSV vaccines.

## Introduction

The direct burden of severe acute respiratory syndrome coronavirus 2 (SARS-CoV-2) infection in children to date has generally been low compared to adults, although this burden appears to be increasing with the spread of the SARS-CoV-2 Delta variant. Measures introduced to slow the spread of SARS-CoV-2 have had several indirect effects on the dynamics of other seasonal diseases that commonly affect children, most notably on respiratory syncytial virus (RSV) and influenza. In this Perspective article, we describe observations for paediatric RSV and influenza in the southern and northern hemispheres during the 2020/21 seasons. We examine their possible trajectories for the coming months, with a specific focus on the temperate countries of the northern hemisphere. We also describe the clinical service implications of the altered disease dynamics for secondary care providers, including preventative prophylaxis for infants at risk. Finally, given these unusual dynamics, we underscore the challenges and opportunities in understanding altered seasonal patterns of respiratory disease, as well as implications for ongoing vaccine trials. We argue that the rapid developments in SARS-CoV-2 molecular epidemiology are readily applicable to new approaches to control influenza and RSV. The future offers an opportunity to strengthen surveillance infrastructure for respiratory viral communicable diseases, in anticipation of the likely introduction of RSV vaccines and better influenza vaccines.

## Impact of the COVID-19 pandemic on the circulation of paediatric respiratory syncytial virus and influenza

Since the emergence of SARS-CoV-2 in Wuhan, China in December 2019, a number of non-pharmaceutical interventions (NPIs) have been implemented in an attempt to slow the transmission of the virus. The NPIs included travel restrictions, closures of workplaces and schools, as well as the widespread introduction of physical distancing, mask wearing, and increased hand hygiene. In addition to interrupting SARS-CoV-2 spread, these implemented measures have had remarkable consequences on the transmission of other respiratory viruses, most notably for RSV and influenza in infants (< 1 year) and young children (< 5 years). In a normal year, RSV causes substantial global morbidity and mortality in children under 5 years of age, resulting in an estimated 3.2 million hospital admissions and 118,200 deaths annually [1] while influenza was responsible for ca 870,000 hospital admissions and 34,800 deaths in the same age group [2].

With COVID-19-related NPIs in effect, incidence of RSV and influenza was altered in the southern hemisphere during the anticipated peak time in winter of 2020 (June–August). A notable reduction in cases and admissions of paediatric RSV and influenza were observed in Australia [3], Chile and South Africa [4], and reduced paediatric intensive care unit (PICU) admissions were seen across South America [5]. Similarly, in the northern hemisphere, intermittent lockdowns also interrupted the spread of RSV and influenza in the autumn/winter of 2020. For example, in England during October and November, no RSV-positive samples were detected in national surveillance. From June 2021 forward, test positivity for RSV and influenza in England’s national laboratory-based surveillance system Respiratory DataMart has remained low, signalling a major reduction in normal seasonal illness attributable to these viral infections [6].

In the absence of a usual autumn/winter seasonal pattern for RSV, countries in both hemispheres have experienced a range of subsequent epidemic trajectories (Table). Four of the five continental Australian states have observed delayed annual winter RSV epidemic peaks. Cases surged during the southern hemisphere spring/summer 2020 period (September–December 2020), with even higher case numbers and admission rates [7] than normally seen during winter periods (June–August). A similar delayed RSV peak was observed in South Africa [8]. In addition, the United States’ (US) Centers for Disease Control and Prevention (CDC) has also reported an increase in RSV activity [9] in May to June 2021, which is unusual for this time of year. Influenza, in contrast, has yet to resurface in Australia, with little or no circulation since April 2020 [10]. Similarly, the US reported in May 2020 that the influenza hospital rates were 90% lower than during the low severity 2011/12 season [11] (Table) with persistently low rates across the autumn/winter of 2020/21. The World Health Organization (WHO) and European Centre for Disease Prevention and Control (ECDC) have reported that influenza activity during the 2020/2021 influenza season did not increase above baseline with no indication of an autumn/winter spike, despite widespread and regular testing for influenza viruses and this has continued into spring and summer of 2021 where influenza activity remained at interseasonal levels [12]. 

**Table:** Seasonal patterns of respiratory syncytial virus since the emergence of SARS-CoV-2 in southern and northern hemisphere countries in 2020/2021 (n = 7)

Country	Hemisphere	Delayed peak? (by June 2021)	Delay in peak (months)	Hospital admission rates compared with previous seasons
Australia	Southern	Yes	6^[^ ^33^ ^]^	Higher^[^ ^33^ ^,^ ^34^ ^]^
South Africa	Southern	Yes	5^[^ ^35^ ^]^	Unknown
New Zealand	Southern	Yes	12^[^ ^36^ ^,^ ^37^ ^]^	Higher^[^ ^36^ ^,^ ^37^ ^]^
France	Northern	Yes	4^[^ ^38^ ^]^	Lower^[^ ^38^ ^]^
United States	Northern	Yes	6^[^ ^39^ ^]^	Lower^[^ ^40^ ^]^
England	Northern	No	NA	NA
Japan	Northern	Yes	7^[^ ^41^ ^]^	Unknown

NA: not applicable; SARS-CoV-2: severe acute respiratory syndrome coronavirus 2.

From a global perspective, the seasonal shift of RSV and influenza raise questions surrounding the patterns of annual re-emergence of viruses from human reservoirs, which may vary between RSV and influenza. For example, the differences in the routes of transmission (direct contact, fomite or aerosol) may explain the differences in seasonal patterns. Now, more than a year after the start of the pandemic, varying degrees of measures against COVID-19 have been imposed in different countries, which might affect the pattern of future annual RSV and influenza epidemics. Thus, an urgent research priority is to understand how these different measures (school closures, infection control measures within schools, and lockdowns) differentially affected patterns of transmission of these respiratory viruses among children.

## Uncertainties around the timing of paediatric respiratory syncytial virus and influenza seasons: potential impact on health services

The delayed epidemiological peaks of RSV and influenza observed in the wake of the COVID-19 emergence has created uncertainties regarding predictions, testing and management of resources. Similar to delayed RSV outbreaks observed in the southern hemisphere, France experienced a delayed RSV peak between January and April 2021, although the number of bronchiolitis hospitalisations at the height of this delayed peak in France appears to be ca 50% lower than observed during the autumn/winter seasons of 2018/19 and 2019/20 [13] (Table). For countries that have experienced an entire year without RSV or influenza, mathematical modelling suggests the possibility of a hard rebound marked by increased morbidity and mortality from one or both viruses in the winter of 2021/22 [14]. However, obtaining information on whether RSV or influenza cases are rising in different countries is complicated by the variation in local testing arrangements, since many countries paused testing for other respiratory viruses to focus on SARS-CoV-2 RT-PCR testing. To meet this challenge, the WHO Global Influenza Surveillance and Response System (GISRS) is adapting to undertake SARS-CoV-2 virological surveillance; while this is necessary for global coordination, it is also an additional burden, particularly in low and middle income countries where influenza and RSV surveillance resources are already stretched. Another factor that may influence unusual RSV dynamics in Europe among infants and children this year includes the reduced rates of preterm birth in some countries [15]. Prematurity is a key risk factor in length of hospitalisation, disease severity, and need for PICU admission [16], with a more than two-fold risk for hospitalisation in preterm infants as compared to term born infants [17].

Why is this uncertainty relevant? A sizeable annual percentage of paediatric hospital admissions (18% of all admissions for infants [18]) and PICU admissions (12% of all admissions for children < 5 years [17]) are usually caused by RSV bronchiolitis. These are usually concentrated in the autumn/winter [17] and this seasonal surge is factored into health service planning. For the northern hemisphere, should current planning account for a possible delayed peak in the summer of 2021? Alternatively, if no peak is seen then, should a potentially large increase in the disease burden caused by RSV in the winter of 2021/22 be expected? In the absence of RSV infections in 2020 and into 2021, a larger cohort of infants and young children than previously remains susceptible [14]. Another consideration is whether winter prophylaxis with palivizumab, a monoclonal antibody given to high-risk infants that reduces the risk of hospitalisation for RSV [19], should be extended beyond the usual administration window in 2021. For influenza, children under 2 years of age comprise one of the most impacted paediatric groups as evidenced by PICU admissions and mortality [20]. If the risk of influenza infection is underestimated this northern hemisphere winter, vaccination rates may fall in both children and in adults. Continued education on the importance of influenza vaccination is needed to maintain vaccine uptake and help to mitigate against potential influenza outbreaks in 2021/22.

## Challenges and opportunities for clinical trials and research

The reduction in the respiratory virus burden in the autumn/winter of 2020/21 will create major challenges for clinical trials and ongoing research. This seasonal irregularity and uncertainty of these infection peaks may affect the execution of new vaccine trials for RSV, or cause delays in the registration of new and improved vaccines for influenza. Currently, numerous Phase II RSV immunisation trials are ongoing in infants and children. While these trials are less likely to be affected, other Phase III trials may be delayed or may need to be postponed pending greater certainty around the timing of RSV peaks in the northern and southern hemispheres. 

Low case counts of RSV hinder the evaluation of primary outcomes of medically attended RSV disease or hospitalisation. Of equal importance, cost-effectiveness analyses of long-lasting monoclonal antibodies such as nirsevimab, recently shown to reduce medically attended RSV [21], will be complicated by uncertainty around the timing of the seasonal burden of infection, and any ongoing physical distancing measures implemented as a result of lack of control of SARS-CoV-2 transmission in the coming years. 

The disruption caused by COVID-19 presents unprecedented research opportunities to study the phylogeographic and transmission dynamics of RSV and influenza following a period of transmission bottleneck. For example, ongoing studies of the molecular epidemiology of samples taken in Australia during the outbreaks in 2020 and 2021 should enable a better understanding of how RSV was re-introduced into communities and spread within and between localities. At present, this is poorly understood, in large part because of the paucity of sequence data and associated epidemiological data worldwide [22]. Key questions that could be addressed are whether there are multiple introductions of the virus (as seen in analyses of influenza [23] or SARS-CoV-2 outbreaks [24]), or seeding events where particular variants cause localised epidemics that spread to neighbouring states or countries. Laboratory and epidemiological data generated by the WHO RSV Surveillance Programme [25] will also provide similar information on a global scale.

How RSV spreads through communities, and whether infection passes through nurseries, schools or workplaces, has not been elucidated. Well-designed research studies would allow for opportunities to examine this, which will help inform future immunisation policies. Whether the SARS-CoV-2 pandemic will affect the global diversity of RSV also has implications for these immunisation programmes, as discussed in more detail below.

For influenza, there may be profound effects on the data available to guide vaccine strain selection, with severe bottlenecks to all influenza lineages (A(H3N2), A(H1N1)pdm09, B/Victoria/2/87-like lineage and B/Yamagata/16/88-like lineage viruses) that were circulating in humans before the SARS-CoV-2 pandemic. The outcome may be the dominance of particular viruses, circulation of a number of variants across all four lineages of influenza, or even the complete disappearance of an influenza type or lineage, such as B/Yamagata/16/88-like viruses, which was on the decline before the pandemic and have been rarely detected since [26]. Careful monitoring of influenza viruses and their circulation, during and after this current pandemic, will be crucial.

Willingness to invest in future respiratory virus surveillance could be harnessed to roll out testing for numerous pathogens at hospital, regional or national level, to enable a broad understanding of the prevalence of SARS-CoV-2, RSV and influenza, and the relationships between these and other respiratory viruses. Interactions between circulating viruses (for example, as observed previously between influenza and rhinoviruses [27]) may cause unusual transmission dynamics and peaks should SARS-CoV-2, RSV and influenza circulate in the same country during the same season.

## Benefitting from the advances made in SARS-CoV-2 research: a new approach to molecular epidemiology for paediatric respiratory syncytial virus and influenza

The response to SARS-CoV-2 has harnessed cutting-edge sequencing technologies, analytical capability and international data sharing platforms, creating an explosion of information about viral diversity (> 2 million whole genome sequences (WGS) deposited in GISAID within an 18-month period, https://www.gisaid.org). In many countries including the United Kingdom, widespread and responsive sequencing of SARS-CoV-2 was integrated into local laboratories and linked to clinical and epidemiological data. With this method, viral genomics can be closely tracked, yielding valuable insights into the effectiveness of population control measures such as vaccine effects on transmissibility, thereby confirming the public health value of a ’sequence-first’ approach. The value of WGS has been highlighted in recent reports from Public Health England about the Delta variant (Phylogenetic Assignment of Named Global Outbreak (Pango) lineage designation B.1.617.2) [28].

This local infrastructure could be leveraged to improve the detection and analysis of circulating RSV and influenza in order to understand interactions between these viruses on a broader level. A standardised system for SARS-CoV-2 lineages was introduced early in the pandemic (github.com/cov-lineages/pangolin) which has helped phylogenetic analyses and outbreak investigations (github.com/artic-network/civet) and this has been further simplified by WHO by assigning Greek letters to variants of interest or of concern (for example, Alpha (B.1.1.7), Beta (B.1.351), Delta (B.1.617.2), and Gamma (P.1)). A similar system for RSV, is currently under discussion and would facilitate large-scale analyses of genomic and epidemiological data by using the proposed universal nomenclature system for RSV below the species level which is already in place [29]. However, an important consideration is the cost and expertise required for high-throughput genome sequencing and associated analyses. Even within the European Union, there are substantial differences in resources, for example, the availability of WGS. In addition, the sizeable costs associated with this approach may not prove sustainable as Europe emerges from the pandemic stage of SARS-CoV-2 circulation.

It also important to emphasise that WGS is just one of a suite of molecular tools that are now available, ranging from the simple and inexpensive MinION sequencing system through conventional Sanger sequencing approaches to a range of Next Generation sequencing platforms. All sequence information, be it a partial, full gene or full genome sequence, contributes to a better understanding of the global evolution and movement of respiratory viruses. More work is needed to establish an understanding of the relationship between viral diversity, antigenic variation, and the basis of escape from either natural or vaccine-induced immunity. This will come from the study of virus isolates in combination with viral sequence data. Firm linking of characteristics of viral phenotype to viral genome will underpin decisions about vaccine strain selection, or the frequency of vaccination campaigns. Adopting similar approaches of combining sequence data with viral isolate characterisation for RSV will yield insights into genotype to phenotype relationships.

Of utmost importance is a well-resourced laboratory infrastructure with capacity to track the response to the widespread administration of monoclonal antibodies to RSV or F-protein-based maternal RSV vaccines, as both approaches rely completely on passive, antibody-based immunity [30,31]. It has already been shown that long-acting monoclonal antibodies are susceptible to failure with antigenic drift in the F protein [32]. Recent SARS-CoV-2 studies demonstrate that a relatively small number of spike glycoprotein mutations (equivalent to the F protein in RSV) may reduce vaccine efficacy [30] or length of immunity following infection; we do not yet know whether the same outcome might prove true for RSV. A baseline understanding of remaining RSV diversity in the post-pandemic era will be important in assessing the likely success of maternal or active immunisations based on the F protein.

## Conclusions

Northern hemisphere countries should prepare for the possibility of a substantial out-of-season RSV peak, and consider implications for hospital management and palivizumab administration. Data sharing and linkage are needed so that surveillance at the national level can be communicated to all local hospitals and clinicians. Most current global data do not contain figures on hospitalisations, which are essential for interpreting the significance of delayed peaks. This is an important surveillance gap. Existing laboratory and research infrastructure should be strengthened to better understand the arrival, nature and transmission of RSV in northern hemisphere countries to better inform the future implementation of RSV vaccines. For influenza, continued surveillance, and support for future vaccination programmes, are needed to prepare for its likely resurgence.
